# Patient Information Summarization in Clinical Settings: Scoping Review

**DOI:** 10.2196/44639

**Published:** 2023-11-28

**Authors:** Daniel Keszthelyi, Christophe Gaudet-Blavignac, Mina Bjelogrlic, Christian Lovis

**Affiliations:** 1 Division of Medical Information Sciences, University Hospitals of Geneva Geneva Switzerland; 2 Department of Radiology and Medical Informatics, University of Geneva Geneva Switzerland

**Keywords:** summarization, electronic health records, EHR, medical record, visualization, dashboard, natural language processing

## Abstract

**Background:**

Information overflow, a common problem in the present clinical environment, can be mitigated by summarizing clinical data. Although there are several solutions for clinical summarization, there is a lack of a complete overview of the research relevant to this field.

**Objective:**

This study aims to identify state-of-the-art solutions for clinical summarization, to analyze their capabilities, and to identify their properties.

**Methods:**

A scoping review of articles published between 2005 and 2022 was conducted. With a clinical focus, PubMed and Web of Science were queried to find an initial set of reports, later extended by articles found through a chain of citations. The included reports were analyzed to answer the questions of where, what, and how medical information is summarized; whether summarization conserves temporality, uncertainty, and medical pertinence; and how the propositions are evaluated and deployed. To answer how information is summarized, methods were compared through a new framework “collect—synthesize—communicate” referring to information gathering from data, its synthesis, and communication to the end user.

**Results:**

Overall, 128 articles were included, representing various medical fields. Exclusively structured data were used as input in 46.1% (59/128) of papers, text in 41.4% (53/128) of articles, and both in 10.2% (13/128) of papers. Using the proposed framework, 42.2% (54/128) of the records contributed to information collection, 27.3% (35/128) contributed to information synthesis, and 46.1% (59/128) presented solutions for summary communication. Numerous summarization approaches have been presented, including extractive (n=13) and abstractive summarization (n=19); topic modeling (n=5); summary specification (n=11); concept and relation extraction (n=30); visual design considerations (n=59); and complete pipelines (n=7) using information extraction, synthesis, and communication. Graphical displays (n=53), short texts (n=41), static reports (n=7), and problem-oriented views (n=7) were the most common types in terms of summary communication. Although temporality and uncertainty information were usually not conserved in most studies (74/128, 57.8% and 113/128, 88.3%, respectively), some studies presented solutions to treat this information. Overall, 115 (89.8%) articles showed results of an evaluation, and methods included evaluations with human participants (median 15, IQR 24 participants): measurements in experiments with human participants (n=31), real situations (n=8), and usability studies (n=28). Methods without human involvement included intrinsic evaluation (n=24), performance on a proxy (n=10), or domain-specific tasks (n=11). Overall, 11 (8.6%) reports described a system deployed in clinical settings.

**Conclusions:**

The scientific literature contains many propositions for summarizing patient information but reports very few comparisons of these proposals. This work proposes to compare these algorithms through how they conserve essential aspects of clinical information and through the “collect—synthesize—communicate” framework. We found that current propositions usually address these 3 steps only partially. Moreover, they conserve and use temporality, uncertainty, and pertinent medical aspects to varying extents, and solutions are often preliminary.

## Introduction

### Background

Summarization is an essential element of human cognition and consists of taking a set of information and retaining the pertinent elements to take action [1]. Feblowitz et al [2] defined information summarization in the health care context as “the act of collecting, distilling, and synthesizing patient information for the purpose of facilitating any of a wide range of clinical tasks.” This definition translates as simplifying the presented information so that health care professionals (HCPs) can act more smoothly and efficiently in different clinical situations.

Automatic summarization of information in electronic health records (EHRs) can serve as a solution for information overload [3], a widespread problem in health care when the presented data are too much to be efficiently processed in a care situation. Information overload can have detrimental effects on patient care in the form of professional stress, fatigue, delays, and medical errors [4]. Although the phenomenon is not novel, it is increasingly present owing to an aging population with an exponentially increasing presence of chronic diseases, increased administrative burden, overabundance, and suboptimal storage of medical data [5,6]. Furthermore, current EHR systems present information in a fragmented manner [7] with widespread repetition, copy-pasting [8], and details not relevant to clinical care [9].

Despite the need for automatic patient information summarization, there is no widely accepted theory or methodology. This report aimed to synthesize the contributions of patient information summarization scattered in the literature. Scoping review is the chosen form with the aim of mapping ideas, mapping concepts related to the question, and identifying knowledge gaps.

The review is not unprecedented: in their narrative review, Pivovarov and Elhadad [10] already summarized the most important contributions to clinical summarization in 2015. Moreover, there have been several published studies surveying the literature in related fields, including the summarization of biomedical literature [11,12], the summarization from medical documents [13], neural natural language processing (NLP) in EHRs [14], named entity recognition, a type of information extraction and NLP technique, free-text clinical notes [15], automatic clinical documentation [16], the visualization of medical information in the clinical context [17-20], the visualization of intensive care unit (ICU) data [21], and the visualization of trends in medical data [22]. The latter reviews, although exhaustive in their specific scope, do not permit the identification of state-of-the-art summarization methods for HCPs. For example, it is difficult to state the current state of research for the management of uncertainty and time in clinical summarization. Moreover, they did not provide any informed guidelines for clinical summarization.

### Objective

This review, building on a broader scope of articles than the combination of all the previous studies, systematically evaluates where, what, and how medical information is summarized; whether summarization conserves temporality, uncertainty, and medical pertinence; and how the propositions are evaluated and deployed.

On the basis of cognitive science literature, this review also proposes a novel “collect—synthesize—communicate” framework to compare studies on how they contribute to clinical summarization.

## Methods

### Overview

The methodology was designed to process a broad scope of articles; hence, different search strategies were combined to diversify the sources. Two reviewers agreed on the selection method: 2 databases with a clinical focus were searched with similar queries and the retrieved articles were filtered by one of the reviewers according to their titles and abstracts. The same reviewer read the remaining reports in the full text and selected them according to the inclusion and exclusion criteria. The same filtering was then carried out on citations within these articles and the citations of these articles. The reporting was done using the PRISMA-ScR (Preferred Reporting Items for Systematic Reviews and Meta-Analyses extension for Scoping Reviews) [23], and a checklist is provided in Multimedia Appendix 1.

The 2 databases searched in this review were the Web of Science Core Collection and PubMed as they contain a broad scope of articles in the medical field and are less inclusive of other articles in computer science, not related to the medical or scientific domain.

The search query for Web of Science was designed as a combination of 2 parts: capturing the summarization process and capturing the health care content. An iterative process was used to define the exact search term, where the gain of adding a keyword was examined by determining whether the first 5% (sorted by relevance defined by Web of Science) of the results from a query containing the new word but excluding the previous words shows any relevant article. This led to the following query: “ALL=((‘ehr’ OR ‘emr’ OR ‘health’ OR ‘patient’ OR ‘medical’ OR ‘hospital’ OR ‘healthcare’) AND (‘summarization’ OR ‘summarisation’ OR ‘summarizing’))” searching in the title, abstract, and metadata of the articles in the database, including the “keyword plus” field containing terms frequently appearing in the body of an article but not mentioned in the title or abstract.

The query in PubMed was “(‘her’ OR 'emr' OR ‘health’ OR ‘patient’ OR ‘medical’ OR ‘hospital’ OR ‘health care’ OR ‘medical record’[(MeSH Terms])) AND (‘summarization’ OR ‘summarisation’ OR ‘summarizing’).” This query was almost identical to the search made in Web of Science except that PubMed does not have a “keyword plus” field like Web of Science to search in. Instead, preindexed articles with the Medical Subject Headings term “medical records” were included in the search.

All results of the queries were imported into the Rayyan app [24], helping to organize the citations for a review article. After duplicates were removed, the abstracts and titles were scanned in this application to filter records that were obviously irrelevant to patient information summarization. The app enables highlighting specific words in the abstract with 2 distinct colors, speeding up the review process. After this filtering step, the remaining articles were read in the full text for inclusion using the inclusion and exclusion criteria detailed in the following section.

After identifying the relevant works, the list of references in the selected articles and the list of citing papers retrieved by Google Scholar were manually reviewed for titles related to the topic. These potentially relevant titles were manually added to the citation manager and further filtered by reading their abstract and eventually reading them in the full text (as with the original results). If these articles contained further (previously unseen) relevant references or citations toward them, they were also processed.

### Inclusion and Exclusion Criteria

According to the inclusion criteria, all records mentioning the summarization of clinical or health data as a general goal in their abstract, proposing solutions for information overflow, or claiming to make steps for these general goals were included.

All records were excluded where the corresponding article was not available in the full text for the authors (ie, at the University of Geneva campus), as the analysis could not be conducted on these records.

As several articles do not mention the source of the data in their abstracts, an exclusion criterion excludes articles about summarizing non-EHR data (eg, summarizing research articles; not EHR). Similarly, articles developing summarization for users other than HCPs *(not for HCP)* or for contexts other than clinical applications *(not clinical)* were excluded.

As many different methods can be labeled as “summarization,” only records presenting a type of overview of a patient’s current or past status are aimed to be included, therefore articles proposing alerts (eg, risk scores and cues) or similar simple parameters to summarize the state of a disease or patient *(alert)* and articles proposing other remedies for information overflow than automatic summarization for information overflow *(not automatic)* were excluded.

As previous reviews analyzed articles using different aspects, a broad timeframe was aimed at the review. However, as early EHR systems were very different from current systems, and hence the concept of summarization is largely different in these systems, articles before 2005 were excluded (*<2005*). The cutoff year is somewhat of an arbitrary (but round) threshold, although contributions before this year are sporadic.

Finally, articles presenting summarization solutions only for nontextual and nonstructured data (eg, video or signal summarization; *Other data)* and review papers *(Review)* were also excluded.

Articles found relevant to the review were evaluated by one of the reviewers for several criteria chosen to answer the following questions:

1. Where is summarization performed?
2. What is summarized? How?
3. How crucial aspects of clinical information are conserved and used?
4. How are the algorithms evaluated?

Textbox 1 presents the detailed criteria. Some of these criteria were defined a priori, whereas others were shaped during the analysis process. For 1 aspect, the input data type for summarization, the analysis was carried out on a broader scope, and reports excluded by the “other data” criterion were also analyzed for this information.

**Criteria according to which articles are evaluated in the analysis part of this review. For some of the criteria, categories are defined a priori. For others, they are shaped during the analysis process (the ones defined a posteriori are marked with an asterisk).**

**General aspects**

- Type of the study
  - Prototype: articles describing a summarization system or algorithm that can be evaluated. The evaluation might be present or absent from the article.
  - Evaluation study: articles evaluating summarization systems, algorithms, or current summarization processes in health care without presenting a new automatic summarization solution.
  - Recommendations: articles with theoretical contributions not being implemented.

**Where are summaries needed?**

- Field of application*: the medical or clinical domain where the summarization is applied. The categories are discovered during the review process.

**What should be summarized?**

- Source of information:
  - Single encounter
  - Multiple encounters
  - This information cannot be inferred from the text.
- Input for the summarization:
  - Structural data: a combination of numerical and categorical data
  - Textual data: free-text patient information present in electronic health record systems

**How to summarize?**

- The summarization method*: the categories of summarization methods are shaped during the review process.
- Presentation*: how the summary is presented to the end user. The types of presentations are shaped during the review process.
- View on the summarization problem:
  - The top-down group represents records where summarization consists of eliminating “disturbances” from all available information, that is, hiding information deemed to be unnecessary.
  - Bottom-up methods see summarization as a process of finding the most salient information available and building a summary from it.

**Aspects to be conserved during summarization**

- Temporality*: if and how temporal information is conserved and used during summarization. The categories are shaped by the discoveries in the scoping review.
- Uncertainty*: if and how the uncertainty of information is represented during summarization. The categories are shaped by the discoveries in the scoping review.
- Medical knowledge*: if any medical knowledge is included in the design of the summarization system or during summarization. The categories are shaped during the review process.

**What is a good summary? Evaluation and deployment**

- Evaluation*: the method of evaluation. The types are shaped according to the discoveries from the review process.
- Deployment: if the summarization system was deployed in real clinical settings

### Collect—Synthesize—Communicate Framework

During the analysis, we developed a new framework to compare methods of how they summarize clinical information. Following the definition presented in the introduction [2], the model divides the summarization process into an ideally sequential process of information collection, information synthesis, and summary communication. Information collection refers to the extraction of information from raw data, synthesis describes the selection and eventual transformation of the retrieved information, and communication refers to the representation of the synthesized information in a human-digestible format.

This view was consistent with that of several sources of cognitive psychology. For example, Johnson [25] describes summarization as a sequence of prerequisites for summarization (including comprehending individual propositions of a story, establishing connections, identifying the consistent structure of the story, and remembering the information), information selection, and formulating a concise summary. This is also similar to the view presented by Hidi and Anderson [26], who discussed selection, condensation, and transformation.

Nevertheless, the few theoretical studies on clinical summarization have slightly different views on the process. Feblowitz et al [2] described clinical summarization as a process of aggregation, organization, reduction, transformation, interpretation, and synthesis. Jones [27,28] describes textual summarization as a process of interpretation, transformation, and text generation. Although these theories mention seemingly different steps for summary creation, they can be mapped to the proposed simpler and more general 3-step framework. Table 1 presents the mapping to the present framework of these theories and some of the most commonly used summarization methods.

**Table 1:** Summary of how existing theoretical frameworks and most abundant summarization methods relate to the collect—synthesize—communicate framework.

Theory or method	Collection	Synthesis	Communication
Feblowitz et al [2]	Aggregation, organization, and interpretation	Reduction, transformation, and synthesis	Organization and synthesis
Jones [27,28]	Interpretation	Transformation	Text generation
Extractive summarization (eg, Liang et al [29])	N/A^a^ (not covered)	Sentence selection	N/A (not covered)
Abstractive summarization (eg, Gundogdu et al [30])	Encoding	Attention mechanism	N/A (not covered)
Topic modeling (eg, Botsis et al [5])	Topic extraction	N/A (not covered)	N/A (not covered)

^a^N**/**A: not applicable.

## Results

### Overview

As shown in Multimedia Appendix 2 [31], a total of 7925 titles were retrieved from PubMed and 3641 articles were retrieved from Web of Science. After removing duplicates, 9166 records were screened by their title and abstract for inclusion criteria and 380 records were chosen for full-text reading. From these, 1 could not be accessed by the authors and 328 were excluded based on the exclusion criteria.

From the 52 articles included in the analysis, 612 records were identified as potentially relevant by their title and 175 titles were chosen to be read in the full text after screening the abstracts. From these 175 titles, 2 could not be accessed, 97 records were excluded according to the exclusion criteria, and 76 titles were included in the analysis.

Among the 128 articles remaining in the analysis, 102 titles were categorized as a prototype, 20 were categorized as evaluation studies, and 6 were categorized as “recommendations.”

### Fields of Application

This review identified diverse fields of application for which summarization methods have been developed. A grouping of these fields is as follows:

1. ICU (27/128, 21.1%), where recent events and vital parameters are summarized
2. *Surgery* (1/128, 0.8%) and *related anesthesiology* (5/128, 3.9%), requiring all the information related to surgery to be summarized
3. *Diagnostics*, showing findings from one or several diagnostic sessions and including radiology (19/128, 14.8%), out of which 5.5% (7/128) were presented as a solution in the MEDIQA 2021 summarization task [32], ultrasound (2/128, 1.6%), prostatectomy (1/128, 0.8%), and laboratory data management in a clinical context (1/128, 0.8%)
4. Hospital care (9/128, 7%), where information related to a hospital stay requires efficient summarization
5. Chronic disease monitoring including diabetes (4/128, 3.1%), HIV (1/128, 0.8%), chronic obstructive pulmonary disease care (1/128, 0.8%), cardiology (2/128, 1.6%), nephrology (1/128, 0.8%), and monitoring of multiple chronic diseases (4/128, 3.1%), where salient events and information during a complex and long-lasting disease are required
6. Oncology (5/128, 3.9%), where the main events and elements of complex treatment are summarized
7. Drug prescription (3/128, 2.3%), where pharmaceutical history is summarized
8. Other medical environments included psychotherapy (3/128, 2.3%), opioid misuse treatment (1/128, 0.8%), general practice (2/128, 1.6%), emergency room (2/128, 1.6%), older adult care (2/128, 1.6%), and maternal care (1/128, 0.8%).

In addition, 25% (32/128) of articles did not specify their field of application or were meant to be usable in multiple types of medical environments and domains.

### Input for Summarization

Regarding the source of information, 62.5% (80/128) of reports talk about systems summarizing single patient encounters, 27.3% (35/128) of reports explicitly talk about summarizing multiple encounters, 6.3% (8/128) of reports implicitly describe multiple encounter summarization, and 3.9% (5/128) of reports did not specify the cardinality of encounters.

**Figure 1:**
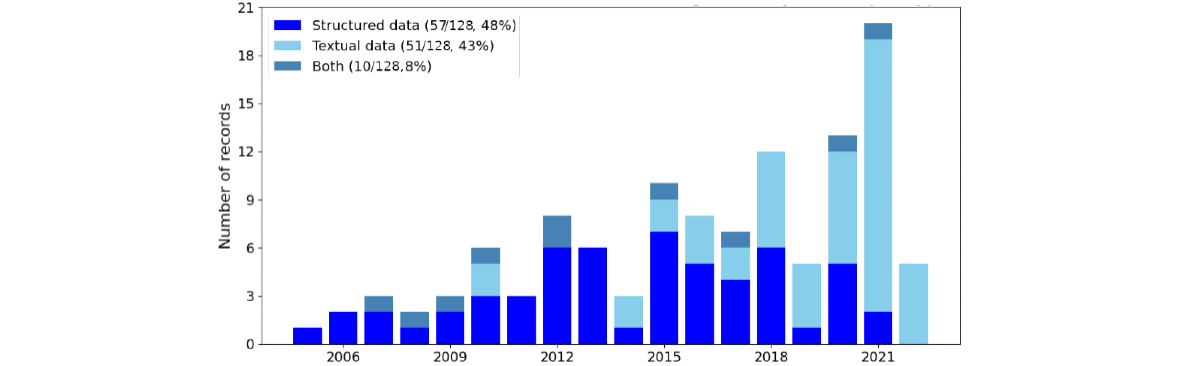
The number of records by year of publication and the input type used in the summarization system or method presented or evaluated. Each column corresponds to a year, the different input types are aggregated into this column, their proportion for the given year is visible on the figure. ICU: intensive care unit.

Among the 128 articles in the review, 3 (2.3%) reports *do not specify the input* type for the summary, 59 (46.1%) worked only with *structured data*, 53 (41.4%) worked only with *textual data*, and 13 (10.2%) worked with *both types*. The trends in the number of articles with different input types are shown in Figure 1. Although more records use only structured data as the input type, the number of articles using textual information has shown a rapidly increasing trend in recent years. Textual information is usually assumed to be in English [33,34] and presents solutions for Finish and German languages [35,36].

A rapid analysis of the excluded articles using other types of clinical data identified the following:

1. Overall, 37 video and image sequence visualization tools in the clinical domain, mainly keyframe extraction [37,38] or motif discovery [39] methods in various fields of medicine, including older adult care, endoscopy [40], hysteroscopy [40,41], laparoscopy [42], magnetic resonance image [39,43], and ultrasound [44]
2. Overall, 20 sensory data simplification techniques using time-series analysis, motif discovery [45], and classification [46] methods for electrocardiogram or other types of signals
3. Overall, 117 articles about summarizing genomic data [47]

### How Data Can Be Summarized? Summarization Methods

Common summarization methods used in the analyzed studies include (a report might use several of these) the following:

1. *Visual design* (59/128, 46.1%) organizes the information visually to help HCPs understand it within a short timeframe.
2. *Concept and relation extraction* (30/128, 23.4%): extracts semantic information from textual information
3. *Abstractive summarization* (19/128, 14.8%) [30,48-52] shortens texts by reformulating them using different wording to describe the content of a document [10].
4. *Extractive summarization* (13/128, 10.2%) [29,53] shortens texts by omitting a part of it, that is, composing a short text (a summary) from extracts of the original document.
5. *Summary specification* (11/128, 8.6%) describes the content to be presented for a summary.
6. *Pipeline extracting* information and *synthesizing and communicating* it with natural language generation tools (7/128, 5.5%)
7. *Topic modeling* (5/128, 3.9%) [54-57] categorizes documents according to their content and labels them with a list of representative words [58]
8. *Time-series analysis* (6/128, 4.7%) identifies characteristic properties in a temporal data series, including motif discovery, identifying meaningful patterns in temporal data (used in the study by Jane et al [59]), trend detection [60], or change detection [61].
9. *Dimensionality reduction* (3/128, 2.3%) treats patient data as a long vector encoding all patient information (ie, a row in a table with many columns), and reduces this information to a shorter vector (ie, a row with a much smaller number of columns) without losing too much information.

Some of these methods are intrinsic to the input data type and work only with a particular data type. For example, time-series analysis (including motif discovery), risk scores, and dimensionality reduction are intrinsic methods for structured data. Although a large number of articles using these methodologies are not included in this review as they are used by non-HCPs (eg, machine learning algorithms), some of the titles propose this approach as the first step to clinical summarization [62-64].

The most common intrinsic methods for textual data are extraction, abstractive summarization, and topic modeling.

Some of these summarization methods can apply machine learning techniques. An overview of the applied machine learning methods is presented in Table 2. The table lists all records obtained using machine learning and categorizes the records according to the summarization method and the type of machine learning method they use. Machine learning methods can be categorized into traditional machine learning methods, deep neural networks, and transformers. Traditional methods include support vector machines, random forests, and conditional random field methods; deep neural networks include deep neural networks, recurrent neural networks, and convolutional neural networks; transformers contain BART [65], BERT [66], Pegasus-based [67] methods, and pointer-generator models. In addition, Reunamo et al [34] used an interpretable machine learning technique (Local Interpretable Model-Agnostic Explanations, LIME [68]). N/A indicates that a machine learning method is not used for a given type of summarization method.

**Table 2:** Summary of records applying machine learning methods for clinical summarization^a^.

	Traditional techniques	Deep neural networks	Transformers
Extractive summarization	SVM^b^+CRF^c^ Liang et al [29], 2019 Liang et al [9], 2021	CNN^d^ Liang et al [29], 2019 Liang et al [9], 2021 Subramanian et al [69], 2021 RNN^e^ Alsentzer and Kim [70], 2018 Liu et al [71], 2018 Chen et al [53], 2019	BERT^f^ Chen et al [53], 2019 Kanwal and Rizzo [72], 2022 McInerney et al [73], 2020 Liang et al [36], 2022 Shah and Mohammed [56], 2020 Other Liang et al [29], 2019 Liang et al [9], 2021
Abstractive summarization	N/A^g^	RNN Gundogdu et al [30], 2021 Hu et al [74,75], 2021	BERT Cai et al [48], 2021 Chang et al [76], 2021 Mahajan et al [77], 2021 Sotudeh et al [50], 2020 BART^h^ Dai et al [78], 2021 He et al [79], 2021 Kondadadi et al [80], 2021 Shing et al [81], 2021 Xu et al [82], 2021 Pegasus Dai et al [78], 2021 He et al [79], 2021 Kondadadi et al [80], 2021 Zhu et al [89], 2021 Xu et al [82], 2021 Pointer generator MacAvaney et al [49], 2019 Zhang et al [51], 2018 Zhang et al [83], 2019 Own architecture Delbrouck et al [84], 2021 GPT-2^i^ Xu et al [85], 2019
Concept and relation extraction	N/A	RNN: Reunamo et al [34], 2022	BART: Tang et al [86], 2022
Pipeline	Random forest: Lee and Uppal [87], 2020	N/A	N/A
Topic modeling	Alternating decision tree: Devarakonda et al [88], 2017	N/A	N/A

^a^Machine learning methods are categorized into traditional machine learning methods, deep neural networks, and transformers.

^b^SVM: support vector machine.

^c^CRF: conditional random field.

^d^CNN: convolutional neural network.

^e^RNN: recurrent neural network.

^f^BERT: Bidirectional Encoder Representation from Transformers.

^g^N/A: not applicable.

^h^BART: Bidirectional Autoregressive Transformer.

^i^GPT-2: Generative Pre-trained Transformer 2.

Summarization methods can also be categorized based on their outputs. The review identified several ways in which the summarized information is presented to the end user:

- A graphical display (53/128, 41.4%) is a specific way (interactive or not) to present information on the computer screen.
- A short textual summary (41/128, 32%) describes information in an ordinary language (eg, English).
- A preset static report: including its content designed to include specific medical information (6/128, 4.7%) or chosen statistical distributions representative of the patient (1/128, 0.8%)
- Problem-oriented view: a view grouping findings according to the problems the patient may present (7/128, 5.5%).
- Low-dimensional vector (4/128, 3.1%): encoding information n numbers (where n is the dimension) where each number represents the state of the patient from a particular aspect.
- List of words representing a topic (5/128, 3.9%), problem list (2/128, 1.6%), list of medical concepts found in the document (2/128, 1.6%), or label (2/128, 1.6%)
- A table (1/128, 0.8%) with rows and columns or a directed graph or concept map representing information in a graph-structured data model (2/128, 1.6%)
- No presentation: the articles in the “recommendation” group (5/128, 3.9%) did not present the results to the end user.

**Figure 2:**
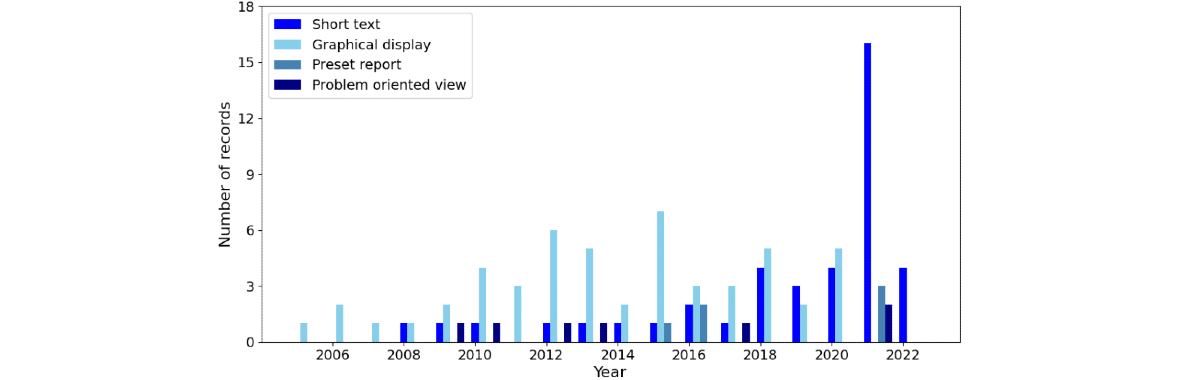
The number of records by year of publication and the most common ways of summary presentation used in the summarization method presented or evaluated in the report.

Figure 2 depicts the evolution (by the time of publication of records) of the most abundant formats for communicating the summarization results.

Concerning their view on summarization, 32.8% (42/128) of the records regarded summarization as a *bottom-up* approach and 64.8% (83/128) used the *top-down* view, whereas 1.6% (2/128) of records *do not show a clear opinion* on summarization.

**Figure 3:**
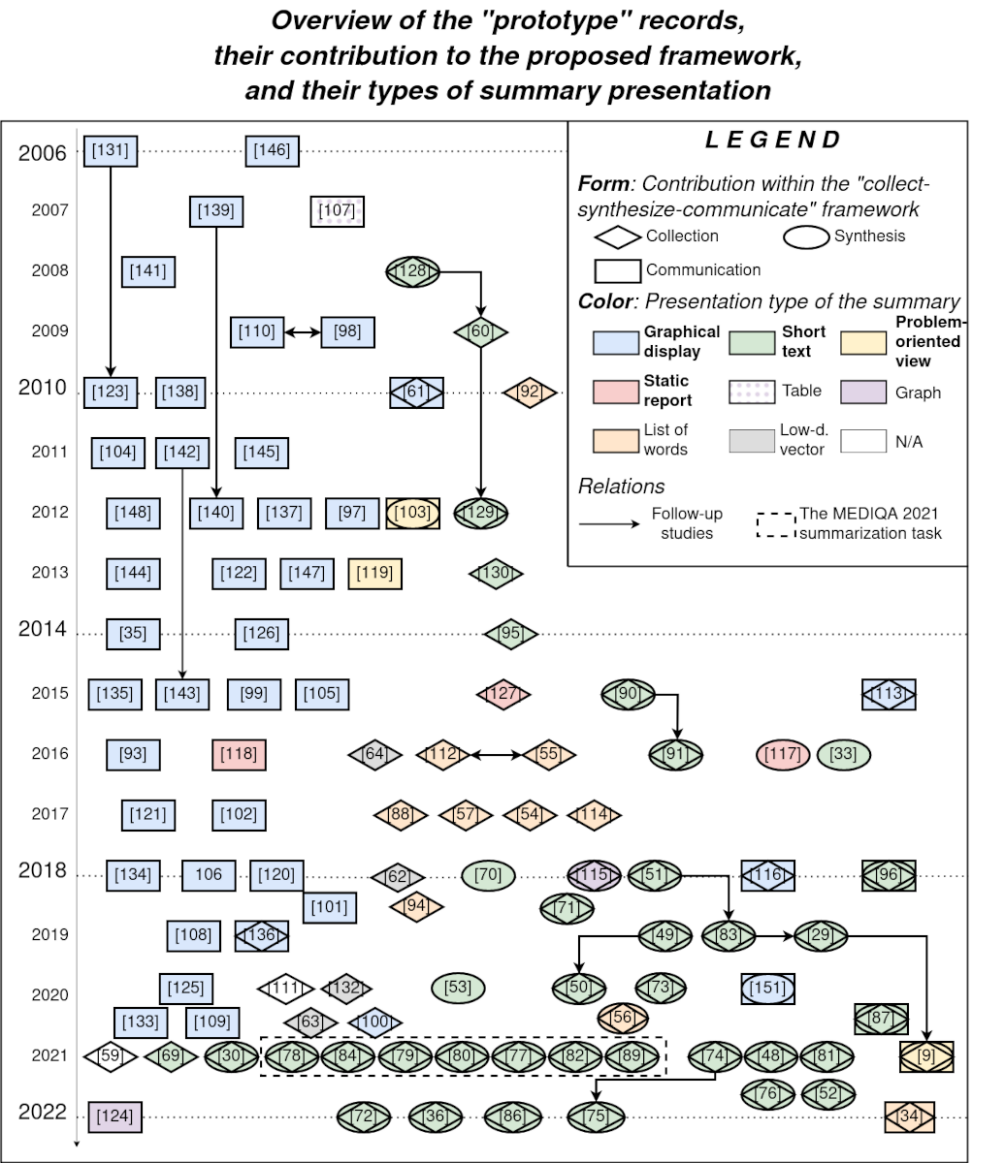
Diagram showing references to all analyzed “prototype” records and how they contribute to the “collect—synthesize—communicate” framework (ie, which step of the framework is addressed within the corresponding work). The records’ year of publication, the presentation of summaries, and the relation between records are also displayed [9,29,30,33-36,48-57,59-64,69-84,86-149].

Using the proposed framework, 42.2% (54/128) of the records contributed to *information collection*, 33.6% (43/128) recorded *information synthesis*, and 46.1% (59/128) presented solutions for *summary communication*. Figure 3 [9,29,30,33-36,48-57,59-64,69-84,86-149] visualizes all the analyzed prototype articles and how they fall into these categories (ie, which step of the framework is addressed within the corresponding work). The records’ year of publication, presentation of summaries, and relationship between records are also displayed. The diagram has a vertical axis showing the year of publication, and all the “prototype” records (presented as a reference) published in that year appear in a line (or in 2 lines if the number of publications for a given year is very high). The order within a line has no significance, although the records were grouped within a line to show their contributions. The shape or shapes surrounding a reference symbolizes the steps that the record addresses in the “collect—synthesize—-communicate” framework. The reference to the study by Liang et al [9] is surrounded by all 3 shapes, indicating that the study addresses all the 3 steps. The records also have a color representing in which format the summaries are presented to the HCPs. Closer relationships (ie, follow-up studies) are also presented. The studies submitted to the MEDIQA-2021 challenge [21] are also marked in the diagram.

Regarding information collection, concept and relation extraction (50/54, 56%), time-series analysis (6/54, 11%), encoding (22/54, 41%), temporal abstraction (6/54, 11%), and topic extraction (5/54, 9%) were proposed as solutions. Medical concepts are extracted from textual data either using publicly available solutions (eg, cTAKES [164] in the study by Goff and Loehfelm [94]) or tools developed by the authors (eg, [113,114,157]). The retrieved list of concepts can be used for simpler tasks, such as problem list generation [88], or some records present systems that take a step further extracting the context [115], syntactic structure [94], or approximate semantic structure of a sentence [116] as well.

With regard to information synthesis, sentence selection by scoring (13/43, 30%), knowledge-based rules (18/43, 42%), and attention mechanism (19/43, 44%) were possible solutions.

Proposals for summary visualizations are usually features on a graphical screen; they are listed and compared in Table 3. For unprocessed textual data, the solutions included highlighting important concepts (3/5, 60%) and creating graphs that visualize the semantic structure of the textual data (2/5, 40%).

**Table 3:** The number of records presenting various features for visualizations in works with graphical displays. A record can use several features.

Feature	Occurrence (out of 58), n
Colors	43
Selection of features	37
Tabular interface	35
Change in time	31
Visualization of divergence	22
Placement of variables	19
Interactive display	18
Pictograms	10
Physical location shown	6
Alerts	5
Size difference	5
Customizability	3
Shape	3
Word cloud	3
Comparison	2
Variability of parameters	1

### Aspects to Be Conserved and Used

In total, 58.6% (75/128) of the titles did not conserve temporal information, whereas 2.3% (3/128) of titles were agnostic to temporal information (they conserve but do not use it). The remaining articles used a variety of approaches:

- *Timeline visualization*: plotting information along a horizontal or vertical temporal axis (34/128, 26.6%).
- *Other visualizations*: showing only the trend of parameters (1/128, 0.8%) or providing a complex visualization framework in which temporal information can be displayed and analyzed (1/128, 0.8%).
- *Information extraction from the temporal domain* by analyzing how the parameters change during the patient journey. This group included a time-series analysis (6/128, 4.7%), pattern recognition (2/128, 1.6%), and change detection (1/128, 0.8%). Time-series analysis (applied in several studies) [59,60,125-127,150] extracts statistical information from the temporal evolution of one or several variables. Pattern recognition [117,128] attempts to identify meaningful patterns in temporal data. Change detection [61] seeks important events that manifest in the trends and patterns of temporal variables. Some studies have revealed the relationship between these events.
- A *theoretical model of temporal events*, which can either describe more complex interactions between temporal events (6/128, 4.7%) or be very simple (eg, creating an order: 1/128, 0.8% or describing events with a single time [n=1]).

It is worth noting that timeline visualization was applied in 3 articles in the temporal information extraction and in 1 article in the complex model of the temporality group as well.

Regarding information uncertainty, 89.1% (114/128) of the articles did not consider the uncertainty of information. Others have proposed the following solutions:

- Statistical methods were used to treat *uncertainty in data*. These methods included correcting detectable errors (3/128, 2.3% [60,79,150]) and optimizing the statistical description of the data using robust statistics (1/128, 0.8% [62]).
- *Uncertainty of temporal events*was described (2/128, 1.6%).
- *Uncertainty of statements* was described by assigning them to uncertainty categories (3/128, 2.3% [71,74,75]) or using existing ontology (3/128, 2.3%).

Medical pertinence was not conserved in 34.4%, (44/128) of the studies (ie, they had no requirements that the summary had any relation to medical concepts or knowledge). A total of 35.9% (46/128) of records used medical knowledge to specify the information to be included in the summary and with what design. Other propositions included the following:

- Using ontologies to find and relate concepts within textual notes (20/128, 15.6% [87,92,93]), the use of Unified Medical Language System (UMLS) extraction tools (6/128, 4.7%) to extract them (eg, [94-96]), or improving the performance of abstractive summarization (2/128, 1.6% [76])
- Use of risk scores to create visualizations (3/128, 2.3% [97-99]) or the application of guidelines to assess risks (2/128, 1.6% [100,101])
- The use of medically salient rules to constrain summarization (3/128, 2.3% [29,61,102])
- Evaluation of the factual correctness of the created summaries integrated into reinforcement learning (2/128, 1.6% [79,83])
- Application of medical knowledge to select pertinent information (2/128, 1.6% [103,151])
- The use of medical knowledge to construct evaluation metrics (2/128, 1.6% [81,152])

### What Is a Good Summary? Evaluation and Deployment

Several types of evaluation methods and metrics are presented in the publications:

- Quantitative measurements in experiments with human participants (31/128, 24.2%)
- Quantitative measurements when summarization was performed in a real clinical environment (8/128, 6.3%)
- Interviews (7/128, 5.5%), focus groups (2/128, 1.6%), or surveys (19/128, 14.8%) asking the opinions of the users after exposure to the summarization system
- Intrinsic evaluation (25/128, 19.5%) of measuring quality by comparing the results to a ground truth
- Performance on a proxy task (ie, disease prediction; 10/128, 7.8%)
- Performance in identifying human-annotated concepts (9/128, 7%) or topics (2/128, 1.6%)

The distribution of the number of human evaluators is shown in Multimedia Appendix 3. Two records [119,173] used significantly more evaluators than other solutions, which are represented as 2 distinct groups at the tail of the histogram. One of these records [173] is a large-scale survey, whereas the other [119] is a pilot study measuring user performance.

Although some records present several evaluation techniques, in 9.4% (12/128) of the articles, no evaluation is presented; in 1.6% (2/128) of articles, the evaluation is not detailed; and in 4.7% (6/128) of records, the evaluation consists of a subjective evaluation carried out by the authors of the article.

The metrics used in the evaluations are as follows:

- *Performance metrics* (eg, precision, recall, and *F* score) on a *prediction/classification task* measuring the “goodness” (validity) of predictions or classifications (used both in usability experiments and formative evaluations; 11/128, 8.6%)
- *Performance metrics* (eg, accuracy) of human participants (ie, the validity of their decisions) on an *experimental task* (24/128, 18.8%)
- *Time savings* due to summarization systems: *time to completion* (*ie, the time needed to perform a predefined task*) in experiments (21/128, 16.4%) or time saved during patient visits (1/128, 0.8%) in deployed systems
- *Patient outcome metrics* (6/128, 4.7%) included mortality and hospital readmission rates.
- The *NASA-TLX score* describes the workload of the user (5/128, 3.9%) and the relationship between the NASA-TLX score and error count (1/128, 0.8%).
- *Number of interactions* (eg, click and screen change) in usability studies (3/128, 2.3%)
- *Grades* given by human evaluators measuring the utility and usability of a system (13/128, 10.2%) or trust in it (1/128, 0.8%).
- Scores comparing textual summaries with properties of the input text. These scores included *Recall-Orientated Understudy for Gisting Evaluation (ROUGE)* [153] (20/128, 15.6%), *bilingual evaluation understudy* [154] (2/128, 1.6%), and *comparison between* input and output *distributions*(2/128, 1.6%).
- *Other properties of the output textual summaries* including *readability/fluency* (10/128, 7.8%), *accuracy* or *factual correctness* (5/128, 3.9%), *completeness* (7/128, 5.5%), and *overall quality* (7/128, 5.5%) in qualitative evaluations of textual outputs. Two (N=128, 1.6%) records distinguished between ontological and nonontological correctness.
- *Proxy measures for the faithfulness* of textual summarization (6/128, 4.7%)
- *Heuristics* derived from requirement specification (5/128, 3.9%)

The evaluation metrics used in quantitative evaluations usually depend on the method of summarization; for dimensionality reduction, it is often a performance metric to predict diseases; for extractive and abstractive summarizations, the ROUGE score [153] is the most commonly used metric, as it is considered the most reliable [32], and for topic modeling, it is its empirical likelihood [174].

For text summarization, evaluations with human participants are often carried out by annotators subjectively grading each produced summary along some metrics, including readability, factual correctness, and completeness. For other summarization methods, this task is usually approximated by either usability tests [134-139] or experiments [140-147] where performance and workload are measured. The few systems deployed in clinical settings are often evaluated by measuring patient outcomes or clinical indicators.

Reviewing the results of each report, some records compared the results with summarization methods in the general domain [48], and 6 (5%) [30,32,50,75,78,144] presented a comparison of clinical summarization methods. The distribution of cross-citations between articles, that is, the number of other publications appearing in the review cited by each report, is represented in Multimedia Appendix 4. Furthermore, 80% of the records cited fewer than 3 other articles analyzed in this review.

Among the 128 records analyzed, 4 (3.1%) talked about a method deployed on a large scale, 7 (5.5%) described deployment in a pilot study, and 1 (0.7%) disclosed the code alongside the publication.

## Discussion

### Principal Findings

#### Where Are Summaries Needed in Health Care?

Publications on clinical summarization are tied to several different medical and clinical fields, mainly where quick decision-making is crucial (eg, ICU) or where a large amount of information is routinely produced (eg, oncology, chronic disease management, and hospital care).

However, some fields requiring quick decision-making (eg, emergency room environments) have seen less progress. In contrast, others where quick decision-making is less critical (eg, radiology) are covered by a relatively large number of records. This discrepancy suggests that clinical summarization can be beneficial in almost all medical fields, although the idea may not have reached all domains at the same pace. Although the previous drivers are easily identifiable, we speculate that the presence of other solutions proposed to handle information overload (eg, the study by Xu et al [85]; see the study by Hall and Walton [4] for review) can decelerate, whereas a shortage of personnel in a field (eg, radiology [155]) can accelerate adaptation.

#### What Should Be Summarized?

The increasing trend in both single-encounter and multiencounter summarizations suggests that both types are salient and should be used depending on the care situation.

Regarding the input for summarization, several experiments show that HCPs can act at least as accurately and in a timely manner with summarized structured [104-110,156] data or textual data [60,157] or with most information coded in these types of data [158] than using complete documentation. Therefore, the focus should be on summarizing textual and structured data when creating summaries for HCPs.

The increasing trend of using textual data for summarization might be attributed to the improvement of NLP, the improved computing power required for some NLP tasks, and the results published by Van Vleck et al [158], who claimed that a significant portion of patient information lies in clinical notes. In contrast, Hsu et al [111] challenged this hypothesis by presenting experiments to predict some clinical measures (eg, hospital readmission and mortality) using textual and structured patient information sources. They concluded that textual sources have little predictive power for the outcomes. However, their analysis might be biased by their methodology, as they use only simple syntactic metrics to describe textual information, whereas semantic information is not included in their model.

#### How Data Can Be Summarized?

The records analyzed in this review show myriad techniques for summarizing clinical data. Some are intrinsic to the input data type and work only with a particular data type, whereas others are not dependent on the input data type.

For textual data, the review reveals more works about abstractive summarization than extractive summarization or topic modeling combined, whereas in the general domain, topic modeling and extractive summarization techniques are the most researched [58,159]. This discrepancy suggests that despite abstractive summarization techniques being immature [160], general problems with extractive summarization, such as redundancy [161], lack of coherence [162,163], and lengthiness [163], can be problematic for clinical applications. The verdict about topic modeling is unclear. Arnold et al [112] argue that clinicians are good at interpreting topic model results, but other records using this technique do not present evaluations with human participants.

An alternative (and natural) way of organizing summarization methods is to assess how they contribute to the summarization process. Motivated by the lack of a widely accepted theory of the summarization process, this review proposes a 3-step (collect—synthesize—communicate) framework to describe the summarization process, where each step should ideally be addressed by all summarization methods.

For the information collection step, many studies assume an easily queriable information source or propose medical concept extraction from textual data as a solution. More complex information (context, syntactic, or semantic structure of statements) is extracted in only a few studies, and some works propose extracting specific aspects as information.

Concerning information synthesis, a common approach is to precisely define the content of the summary (eg, [118,165,166]) or at least its format [167]. However, these studies do not evaluate the quality of their proposition (except the study by Ham et al [119]). In contrast, some records carried out experiments on the information needs of physicians [158,168]; however, the results were not integrated into any of the reviewed systems.

Concerning summary visualizations, there is no clear opinion on whether textual or graphical summaries are preferable in the medical context. Although there is a slight dominance of graphical displays among the analyzed records, some works [169,170] argue that textual summaries lead to more accurate decisions. However, a general pattern of these works is that they compare a specific graphical display with a particular textual display, limiting generalizability. These contradictory results suggest that both formats are helpful for clinical summarization, if relevant features are present. Problem-oriented views presented in some records (eg, [120]) can include both types of display and might have other advantages, as they group all available information about patient-specific problems [171].

Concerning the view on summarization, both top-down and bottom-up approaches are justifiable in a clinical setting. However, several bottom-up approaches have been inspired by studies that use top-down approaches. One example is the recent development of techniques for identifying salient concepts in source documents for abstractive summarization. This phenomenon may be due to the natural need for accountability and interpretability, which can be achieved more easily with a bottom-up approach closer to human cognition. The need for bottom-up approaches also suggests that there is a need that summarization techniques address all 3 steps of the proposed “collect—synthesize—communicate” framework, including information collection, synthesis, and visualization.

#### How Are the Temporal, Uncertain Aspects of Information and Its Medical Persistence Conserved and Used?

The temporal nature of clinical data is an essential aspect of clinical reasoning [172], and a relatively large portion of analyzed records presents solutions to use this aspect of information. However, in most of these studies, this aspect was only represented as a visualization feature. Most visualizations are timeline visualizations, plotting information along a horizontal or vertical temporal axis following Plaisant et al [175], although some alternative methods exist [121-124]. Alternatively, some studies have revealed the relationship between events by analyzing how variables change during the patient journey.

Although temporality in clinical settings is believed to be more complex [172] than a series of punctual events, current solutions to clinical summarization hardly reflect this complexity. Very few studies have attempted to incorporate more temporal information by using more complex models of temporality (eg, events lasting during an interval). Complex temporal information is usually not directly available in patient records and must be deduced from the context and knowledge-based rules. This process is called temporal abstraction and was applied in previous studies [90,91,129-132,176]. Hunter et al [129,130] considered the uncertainty of temporal information by defining the beginning and end of each time interval as an interval.

Although several levels of uncertainty exist in clinical care [177], the majority of the analyzed reports do not present solutions to conserve or handle any information uncertainty.

To a lesser extent, the pertinence of medical knowledge has been overlooked by many summarization approaches. Many records do not consider medical pertinence or use it only for some design considerations. However, the few records that handle this aspect of medical data provide a relatively wide range of solutions to constrain the resulting summaries. In most cases, these constraints are relatively weak; for example, concepts are assumed to be part of a specific medical ontology. This is obviously the case for concept extraction tools, but the records using reinforcement learning approximate factual correctness using this approach as well.

Deeper integration of medical knowledge is only present in works using medical rules to select salient information and in the 2 works using medical rules to create summaries. Liang et al [9] used medical knowledge to create components of their proposed NLP pipeline, whereas Shi et al [102] used medical knowledge–based rules to visualize abnormalities in the human body.

#### How to Identify a Good Summary?

Using the definition of clinical summarization (ie, simplifying and presenting information so that HCPs can act more smoothly and efficiently in different clinical situations), the ultimate purpose of an evaluation might be to determine whether using the proposed summarization systems would improve the efficiency of HCPs.

However, such an evaluation is often unfeasible owing to the high costs and ethical issues associated with potential medical errors.

This is supported by the results, as many of the proposed evaluations are approximative solutions, and there is quasi-uniform agreement that evaluating summarization is challenging and suboptimal [168]. The spectrum of these evaluations is broad, but a common trend is to carry out a qualitative evaluation using an easily calculable evaluation metric describing either the quality of the summary or its “usefulness” to perform a proxy task (ie, disease prediction).

These qualitative analyses are suboptimal. For example, one of the most common qualitative metrics, the ROUGE score, assumes a human-annotated “gold standard” summary to which to compare, but this standard may not exist given the high cost of annotation or because there are disagreements between people about what would be a “gold standard summary” [70,168]. To tackle this problem, some records [72,178] present a comparison between the semantic distribution of the input and the summary, whereas others [133,150,179] use heuristics to evaluate the results. Another problem with the ROUGE score is that even with a high ROUGE score, a summary can be very inaccurate [168]; therefore, there have been attempts to measure the “faithfulness” of summaries either by the number of medical concepts retrieved [81] or with a more complex faithfulness measure defined by Zhang et al [83].

Evaluations with human participants often complement the qualitative evaluations. Human evaluations have mainly positive outcomes (except in the study by van Amsterdam et al [148]); however, most of the evaluations are carried out on a small scale. This can explain why very few long-lasting implementations in health care have been presented in the literature.

It is also important to mention that there is very little comparison between summarization methods, and citations between records are scarce. This suggests that the research in this domain is fragmented.

These shortcomings suggest that evaluation is a weak point in clinical summarization proposals, and the lack of widely accepted evaluation metrics and methodology might be a main obstacle for research in the field.

### Limitations

Methodological biases are present in selection, synthesis, and reporting. First, the number of reviewers was limited both in the selection and analysis of records, resulting in selection and synthesis bias.

Selection bias also comes from the fact that the review was carried out on works published in a scientific paper or in the gray literature, and the initial search was carried out on 2 databases that are more specific to medicine. However, several unpublished summarization solutions have been applied to current EHR systems.

Moreover, publishing bias also adds to selection bias, as there is a clear dominance of positive results in scientific publishing.

Furthermore, the applied research queries and the selection of the database are also a source of bias. Using other data sources (eg, IEEE Xplore and Scopus) might have introduced further bias to the analysis. However, including the citations and references in the review process might have reduced this bias significantly. Moreover, the queries are formulated in English; therefore, results not in English and containing non-English terms might be missed if their abstract was not translated or if they do not appear in the citation list or references in the retrieved articles. Finally, there were potentially relevant records [149,180,181], where the full text could not be read and analyzed as it was not available at the time of writing the manuscript.

### Conclusions

Clinical summarization has not reached all domains at the same pace, although it is potentially beneficial in most medical fields. Two aspects, the requirement for quick decision-making and the overabundance of data, were identified as the main drivers for the development of automatic summarization methods. However, other less-evident drivers might also play a role in adaptation.

Despite this need, very few [113,119,182] scientific publications are presenting adaptation in real clinical settings, suggesting a low success rate in clinical environments.

Despite the large number and variety of propositions, hardly any comparisons exist between the solutions. This low rate is due to the difficulty in comparing the summarization methods.

From a cognitive psychological perspective and to measure how the summarization methods align with the definition of summarization, this review proposes to compare these algorithms through a “collect—synthesis—communicate” framework referring to information gathering from data, its synthesis, and communication to the end user.

Only a small proportion of the current propositions address all 3 steps, and none of the most abundant methods (ie, abstractive, extractive summarization, and visual design) address them completely.

Beyond the lack of alignment of the dimensions of summarization, propositions conserve and use crucial aspects of information (temporality, uncertainty, and medical pertinence) to varying extents.

Although uncertainty is rarely considered, temporality and some medical pertinence are conserved during some presentations, but the solutions are often preliminary or lack depth in these aspects. Further research is necessary to address these issues.

Nevertheless, the main shortcoming of the current automatic summarization methods is the lack of consistent evaluation. Although there are some new proposals to evaluate the quality of summarizations more rigorously [83], further research is required to relate these metrics to human perceptions.

## Appendix

Multimedia Appendix 1 PRISMA-ScR (Preferred Reporting Items for Systematic Reviews and Meta-Analyses extension for Scoping Reviews) checklist for the review.

Multimedia Appendix 2 PRISMA (Preferred Reporting Items for Systematic Reviews and Meta-Analyses) 2020 flow diagram describing the review process applied in the study [182].

Multimedia Appendix 3 Histogram showing the distribution of the number of evaluators in studies where evaluations with human participants are present.

Multimedia Appendix 4 Histogram showing the number of other publications appearing in the review cited by each report. The number of cross-citations between the works are low.

## Abbreviations

EHR: electronic health record
HCP: health care professional
ICU: intensive care unit
LIME: Local Interpretable Model-Agnostic Explanations
NLP: natural language processing
PRISMA-ScR: Preferred Reporting Items for Systematic Reviews and Meta-Analyses extension for Scoping Reviews
ROUGE: Recall-orientated Understudy for Gisting Evaluation
UMLS: Unified Medical Language System
