# Supplementary material for: Patient Information Summarization in Clinical Settings: Scoping Review
Source: JMIR Med Inform. 2023 Nov 28;11:e44639. doi: 10.2196/44639 (PMC10716777; doi:10.2196/44639)
Supplement: Multimedia Appendix 2 [file medinform_v11i1e44639_app2.docx]

**Identification of studies via citations (in/out)**

**Identification of studies via databases and registers**

Records identified from:

Citation searching

(n = 612)

Records removed *before screening*:

Duplicate records removed (n = 2400)

Records identified from*:

PubMed (n = 7925)

WebOfScience (n = 3641)

**Identification**

Records screened

(n = 9166)

Records excluded

(n = 8786)

Reports not retrieved

(n = 2)

Reports sought for retrieval (n = 175)

Reports sought for retrieval (n = 380)

Reports not retrieved

(n = 1)

**Screening**

Reports excluded:

Not automatic (n=34)

Not EHR (n = 20)

Not clinical (n = 15)

<2015 (n=20)

Alert (n=2)

Other data (n = 6)

Reports excluded:

Not EHR (n = 79)

Not clinical (n = 78)

Not automatic (n=37)

<2015 (n=20)

Alert (n=5)

Not for HCP (n=9)

Other data (n = 168)

Reports assessed for eligibility (n = 379)

Reports assessed for eligibility (n = 173)

**Included**

*Figure 1 : The PRISMA 2020 flow diagram describing the review process applied in the paper based on*  Page MJ, McKenzie JE, Bossuyt PM, Boutron I, Hoffmann TC, Mulrow CD, et al. The PRISMA 2020 statement: an updated guideline for reporting systematic reviews. BMJ 2021;372:n71. doi: 10.1136/bmj.n71.
