# Supplementary material for: Patient Information Summarization in Clinical Settings: Scoping Review
Source: JMIR Med Inform. 2023 Nov 28;11:e44639. doi: 10.2196/44639 (PMC10716777; doi:10.2196/44639)
Supplement: Multimedia Appendix 3 [file medinform_v11i1e44639_app3.docx]

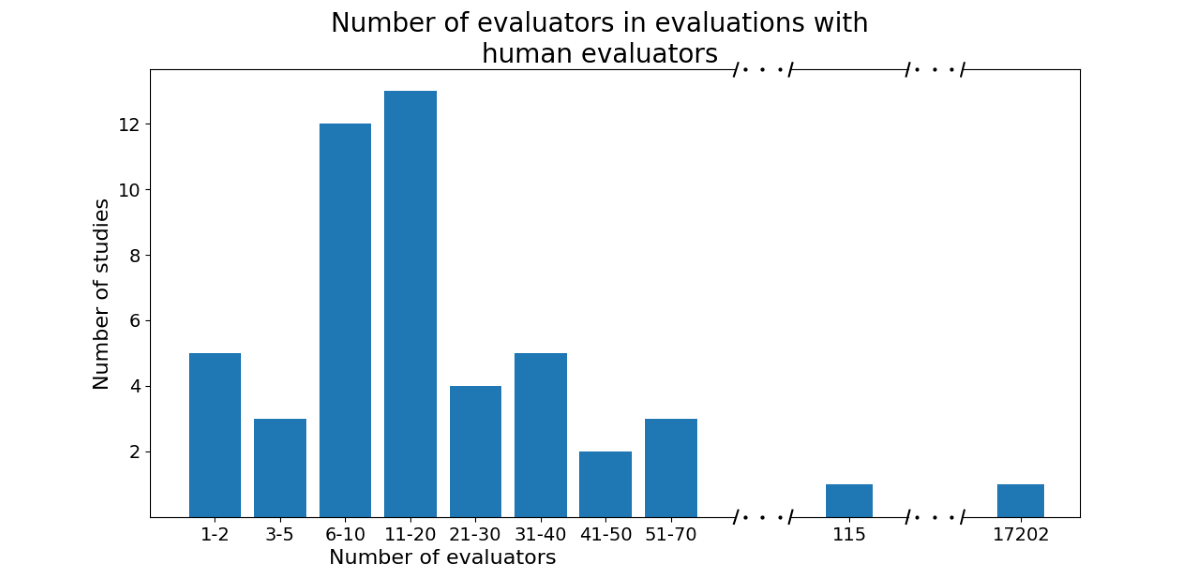
**Figure 1**: Histogram showing the distribution of the number of evaluators in studies where evaluations with human participants are present.
