# Supplementary material for: Patient Information Summarization in Clinical Settings: Scoping Review
Source: JMIR Med Inform. 2023 Nov 28;11:e44639. doi: 10.2196/44639 (PMC10716777; doi:10.2196/44639)
Supplement: Multimedia Appendix 4 [file medinform_v11i1e44639_app4.docx]

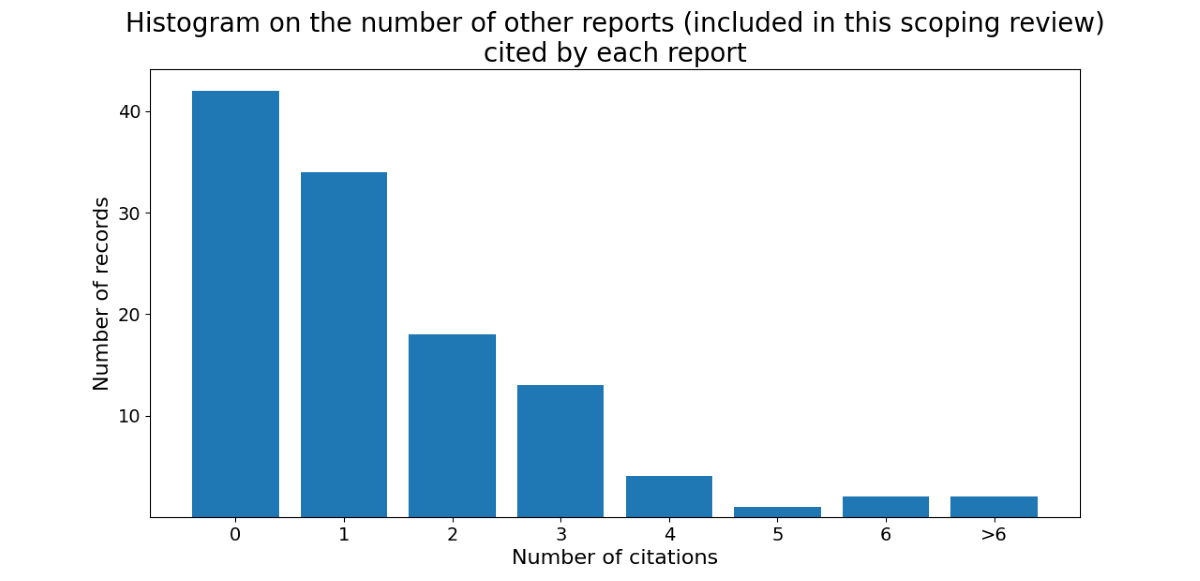


**Figure 1**: Histogram showing the number of other publications appearing in the review cited by each report. The number of cross-citations between the works are low.
